# The selection of search sources influences the findings of a systematic review of people’s views: a case study in public health

**DOI:** 10.1186/1471-2288-12-55

**Published:** 2012-04-20

**Authors:** Claire Stansfield, Josephine Kavanagh, Rebecca Rees, Alan Gomersall, James Thomas

**Affiliations:** 1 Evidence for Policy and Practice Information and Coordinating (EPPI-) Centre, Social Science Research Unit, Institute of Education, University of London, London, UK; 2 Centre for Evidence and Policy, King’s College London, London, UK

## Abstract

**Background:**

For systematic reviews providing evidence for policy decisions in specific geographical regions, there is a need to minimise regional bias when seeking out relevant research studies. Studies on people’s views tend to be dispersed across a range of bibliographic databases and other search sources. It is recognised that a comprehensive literature search can provide unique evidence not found from a focused search; however, the geographical focus of databases as a potential source of bias on the findings of a research review is less clear. This case study describes search source selection for research about people’s views and how supplementary searches designed to redress geographical bias influenced the findings of a systematic review. Our research questions are: a) what was the impact of search methods employed to redress potential database selection bias on the overall findings of the review? and b) how did each search source contribute to the identification of all the research studies included in the review?

**Methods:**

The contribution of 25 search sources in locating 28 studies included within a systematic review on UK children’s views of body size, shape and weight was analysed retrospectively. The impact of utilising seven search sources chosen to identify UK-based literature on the review’s findings was assessed.

**Results:**

Over a sixth (5 out of 28) of the studies were located only through supplementary searches of three sources. These five studies were of a disproportionally high quality compared with the other studies in the review. The retrieval of these studies added direction, detail and strength to the overall findings of the review. All studies in the review were located within 21 search sources. Precision for 21 sources ranged from 0.21% to 1.64%.

**Conclusions:**

For reducing geographical bias and increasing the coverage and context-specificity of systematic reviews of people’s perspectives and experiences, searching that is sensitive and aimed at reducing geographical bias in database sources is recommended.

## Background

### Theory on comprehensive searching for research about people’s views in public health

Systematic reviews of people’s views, understandings, beliefs and experiences (‘views studies’) are valuable to policy-makers in providing contextual information on interventions to inform their development, implementation and evaluation [1]. We describe ‘views studies’ as those that are centred on people’s own voices; these are often qualitative, but not always [1]. Undertaking a systematic literature search for these studies contributes to the rigour and quality of the review findings, but the process of identifying research on people’s views can be challenging. Studies on people’s views tend to be dispersed across a range of subject disciplines, are diverse in their terminology, and exist in various publication formats. People’s views of public health issues potentially cross over the fields of social science, the environment, health and medicine, education and psychology. They are contained across a range of literature search sources from large ubiquitous databases to smaller specialised datasets focused on specific subject areas. Furthermore, there is large variation in the terminology used to describe research methods and in the database indexing of relevant literature [2-4]. Although many studies are published as journal papers, a significant proportion are disseminated in research reports, books, theses and conference proceedings [5].

As many systematic reviews are commissioned to provide evidence for policy decisions in specific geographical regions, there is a particular need to minimise regional bias when seeking out research studies. Gomersall and Cooper [6] highlight the potential bias through selecting large US-based medical databases to seek out information for UK-policy relevant reviews in social science. Although there is no guarantee that searching a wider range of databases increases the percentage of relevant papers identified, failing to consider the breadth and geographical representativeness of the databases selected raises concerns about evidence that could have been missed. We describe this concept as ‘database selection bias’.

We are not aware of research published on database selection bias that relates to the selection of search sources and its effect on the findings of a systematic review. Song et al. [7] classify a range of biases associated with publishing and identifying research in reviews of health care effectiveness and related areas. They refer to geographical bias in the context of database-indexing and observe that some databases contain a predominance of journals from specific geographical regions [7], p35. Howes et al. [8] reflect on biases relating to searches within the public health literature. Other authors have assessed search sources utilised for systematic reviews within the broad field of public health and social care [9-11], and there are other case studies of search sources used in systematic reviews of qualitative studies in public health [12] and education [13]. However, none of these examine the impact of database selection bias on the findings of a review of people’s views.

There is no clear consensus on the methods used to locate views studies for systematic reviews. In seeking views studies in public health, researchers at the Evidence for Policy and Practice Information and Co-ordinating Centre (EPPI-Centre) search across a range of sources using many free-text and controlled vocabulary search terms. The approach attempts to find all relevant research studies. However, this idealistic concept is limited by practical constraints, such as access to resources, time invested in developing the search strategy and searching for studies, and knowledge of both appropriate search sources and search terms.

### Applying theory to practice – literature searching undertaken for a systematic review of people’s views

We now present results from a retrospective analysis of search sources used for a review of children’s views about obesity, body size, shape and weight [14]. The review was undertaken to help inform policy development at the UK Department of Health in relation to children and obesity. It drew on both qualitative studies and other study designs, and sought studies based in the UK in order to maximise its relevance to UK policymaking.

The literature search for the review involved: carrying out extensive and sensitive searches of nine bibliographic databases (using controlled vocabulary and many free-text terms) from a range of disciplines; handsearching of three journals, 16 websites and reference lists; contacting authors and other key informants; and forward citation checking (looking for studies that cite studies included in the review). The database searches were based upon one or more of the following three concepts: body size; children; and people’s views or qualitative study designs. These were limited to English language studies published from 1997 until the date of searching, which was during June and July 2008. A draft search strategy was detailed in a review protocol, which was circulated to the advisory group of the review and made publicly available on the EPPI-Centre’s website. Feedback from AG in the advisory group prompted supplementary searches of seven additional sources that could potentially contain more UK-based studies. The search sources are presented in the Results section. The full search strategy is detailed within the published review [14], on pages 131–139.

Search results were uploaded into a review management software tool (EPPI-Reviewer 3.0) [15] and duplicate papers of the same record were removed. The remaining reports were screened on the basis of their title and abstract, and relevant items were then screened on the full-text of the paper/report. Twenty-eight studies were entered into the review’s syntheses. Study quality was appraised using criteria adapted from Shepherd et al. [16], and is explained in the published review. This focused on two areas: i) the reliability of study findings (based on the rigour of study methods including sampling, data collection and data analysis; and whether findings appeared grounded in or supported by data); and ii) the usefulness of study findings (based upon the breadth and depth of findings as well as whether there was evidence of attention by researchers to an ideal of privileging young people’s views [1,14]).

Two separate syntheses were conducted: one interpretive, which developed overarching themes from the study findings [17]; and one aggregative, which identified similarities and differences between studies addressing similar research questions. Thematic codes were applied to each of the studies included in the interpretive synthesis. An Excel spreadsheet was used to capture the questions addressed by studies in the aggregative synthesis.

### Aims and research questions

The aim of this case study is to examine the impact of database selection bias on the findings of a review of UK children’s views of body size, shape and weight. Our research questions are: a) what was the impact of search methods employed to redress potential database selection bias on the overall findings of the review? and b) how did each search source contribute to the identification of all the research studies included in the review?

## Methods

The impact of using additional search sources to minimise database selection bias was assessed by determining how many new studies were located, and how the studies influenced the findings of the systematic review. Search source data for the studies included in the review were obtained from EPPI-Reviewer 3.0 [15], which contains a record of where each study was found. Where multiple papers had been published about the same study, the paper (and, by association, its source) identified first was selected to represent the study. The impact of the five additional included studies [18-22] on the review findings was assessed by retrieving the qualitative themes (and codes) applied to each study, along with the related extract of the study text. The contribution of each of these studies to the synthesis was assessed by counting how many distinct themes had been attached to that study and comparing this with the themes assigned to the other studies [23]. A note was also made of the quality rating given to each study. To explore the contribution of the types of search sources used to locate studies, the sources were grouped into those that: were in the original protocol; and those that were introduced into the search strategy after the first draft of the protocol had been reviewed. Where possible, the relevance of the retrieved records from each search source, expressed as precision, was determined as the proportion of studies included, compared with the total identified from the search query.

## Results

This section details our findings according to our two research questions: the first examines the impact of the additional search sources on the findings of the systematic review; the second section describes all the search sources and how each contributed to the number of studies judged to be relevant for answering the review question.

### Research question 1: What was the impact of search methods employed to redress potential database selection bias on the overall findings of the review?

Table 1 summarises the distribution of included studies among sources split from the original search protocol and supplementary sources searched later to address geographical bias. While most of the studies were found from the original protocol, over a third of studies were also identified in the later sources and nearly a fifth were only found within these sources. Three of the seven sources used in the supplementary searches provided five studies that were not found by other means. Table 2 shows these five studies, their quality ratings and their contribution to the themes within the review. In terms of quality, three of the five studies were judged to have findings that were highly reliable. In the review as a whole, only two further studies were awarded this quality rating. All of the five studies contributed to the review’s syntheses: one contributed to the interpretive synthesis (of the overarching themes in children’s discussions of body size); and four contributed to the aggregative synthesis (of similarities and differences between studies).

**Table 1:** Distribution of included studies across search source groups

**Search source type and stage of searching in protocol**	**Search sources**	Included on full-text (N = 28)*	**Studies only found within this group of sources (% of 28 studies)**
Bibliographic database search sources in original search protocol	Applied Social Sciences Index and Abstracts (ASSIA); Bibliomap; Cumulative Index to Nursing and Allied Health Literature (CINAHL); Education Resources Information Center (ERIC); HealthPromis; PsycINFO; PubMed; Social Sciences Citation Index(SSCI); Dissertation Abstracts;	16 (57%)	9 (32%)
Search engines in original search protocol	Google and Scirus	3 (11%)	1 (3.5%)
Non-database sources in original protocol	Author contact; forward citation checking (Web of Knowledge); reference list checking; previous EPPI-Centre review; 16 websites; handsearches of 3 journals	8 (29%)	6 (21%)
Supplementary search sources	British Education Index; Child data; IBSS, Index of British Theses; Social Care Online; (The British Library Integrated Catalogue and Zetoc were also searched but did not yield additional studies)	10 (36%)	5 (18%)

*total exceeds 28 as some included studies were identified in multiple categories.

**Table 2:** Assessing the Impact of additional five studies on the findings in the review

**Study**	**Search source**	**Reliability of findings**	**Usefulness of findings**	**Contribution to themes within the review synthesis** **(No. of other studies contributing to the theme)**
**Aggregative synthesis**				
Currie et al. [20]	Child data	High	Low	1 theme:
				Satisfaction (12)
Currie et al.[18]	Child data	High	Low	1 theme:
				Satisfaction (12)
Harris [21]	Index of British Theses	Medium	Low	5 themes:
				Estimations (8)
				Preferred size (9)
				Satisfaction (12)
				Stereotyping (6)
				Importance/concern (2)
Waterston [22]	Index of British Theses	Medium	Low	4 themes:
				Estimations (8)
				Preferred size (9)
				Satisfaction (12)
				Other – relation between self-esteem and experience of size-related bullying (1)
**Interpretive synthesis**				
Stewart et al. [19]	IBSS	High	Medium	3 themes:
				Size matters later (3)
				Diet and exercise as influences (9)
				Appropriate strategies (5)

The single study by Stewart et al. [19] that contributed to the interpretive synthesis contributed to three of the 17 themes identified in this synthesis. It is possible that without this study, one theme (‘size matters later’) would have been relegated to a passing mention within a higher order theme, or might not have been considered at all. Only in Stewart et al. [19] was the idea of adult and children’s experiences of food and size interpreted as an important theme by the authors. In each of the other two studies coded with this theme, the reviewers had mentioned age and body size within an individual quote, but the studies’ authors had not made this connection. The other two themes in Stewart et al. [19] (‘diet and exercise as influences’ and ‘appropriate strategies’) were developed using findings from four and eight other studies, respectively.

The four additional studies that were placed in the aggregative synthesis contributed, in total, to six of this synthesis’ seven themes. All of these themes were, however, usually supported by findings from at least five other studies that had been found with the original searches. While the findings may have looked relatively similar if these studies had not been found, the retrieval of these four additional studies added breadth, depth and strength to this synthesis, thus increasing the quality and rigour of the findings

### Research question 2: How did each search source contribute to the identification of all the research studies included in the review?

Table 3 shows the utility of the 25 search sources in finding studies for the systematic review. Studies judged potentially relevant on title and abstract were found in all search sources (data not shown). The 28 studies ultimately included in the review were located in 21 search sources (this includes duplicate papers found in more than one location). The largest sources of studies were among three international medical and social science databases: Pubmed = 8 (15%); PsycINFO = 7 (13%); and the Social Sciences Citation Index (SSCI) = 7 (13%). While these figures include duplicate records of the same study, over a third of the studies in the review were identified from these three sources (11/28 = 39%). Other databases each identified three or fewer studies. Six studies were found only using non-database sources (author contact, website handsearching, a previous EPPI-Centre review and reference checking). One study was found only from the search engine Scirus. Seventeen of the 28 studies were located in one place (unique studies) and these were spread across 12 search sources, with no single source identifying more than two unique studies. Precision ranged from 0.21% for ERIC to 1.64% for the British Education Index. In terms of overall performance, the Pubmed search yielded the most records to screen (3697) and had a precision of 1.57%, but provided the highest number of studies included within the review, including two unique studies.

**Table 3:** Utility of search sources in identifying relevant studies

**Search source**	**Records retrieved from search**	**Studies included from that source (% precision)**	**Studies included that were unique to that source**
Applied Social Sciences Index and Abstracts (ASSIA)	625	3 (0.48%)	1
Author contact (n ≥ 50)	n/a (12)	2	2
Bibliomap (EPPI-Centre health promotion research register)	16	1 (6.25%)	
British Education Index	61	1 (1.64%)	
British Library Integrated Catalogue	n/a (9)		
Child data	561	2 (0.36%)	2
Cumulative Index to Nursing and Allied Health Literature (CINAHL)	1703	2 (0.11%)	
Dissertation Abstracts	451	1 (0.22%)	1
Education Resources Information Center (ERIC)	468	1 (0.21%)	1
Forward citation checking (Web of Knowledge)	88	1 (1.14%)	
Google	100*	2	
Google Scholar	300*		
HealthPromis (UK Health Development Agency research register)	235	2 (0.85%)	
Index of British Theses	n/a (16)	2	2
International Bibliography of the Social Sciences (IBSS)	402	5 (1.24%)	1
Journal hand searching (3 journals)	n/a (13)		
Previous EPPI-Centre review [24]	n/a (13)	2	2
PsycINFO	1691	7 (0.41%)	
Pubmed	3697	8 (0.22%)	2
Reference checking of included studies	n/a (20)	1	1
Scirus (scientific research search engine)	200*	1	1
Social Care Online	150	1 (0.67%)	
Social Sciences Citation Index (SSCI)	1915	7 (0.37%)	
Website hand searches of 16 sites	n/a (8)	2	1
Zetoc (British Library’s electronic table of contents)	n/a (12)		
**TOTAL**	**12766**	54 studies**	**17** studies**

n/a = screening was offline and a complete record of total items retrieved and screened is not available.

*The quantity screened from a larger list of search results, therefore precision is not calculated.

**54 studies from 61 research records, which following removal of duplicates were 28 studies of which 17 studies were unique to one source.

## Discussion

The five studies identified through the additional searches had an important impact on the review findings; indeed, one study had a central role in the development of one of the review’s descriptive themes. The other four studies were less influential, but added detail and strength to the review’s findings. If this group of additional studies had not been found, the syntheses would have been a less complete story of children’s views of body size. These five studies were also of a disproportionally high quality compared with the studies in the review as a whole and so added to the robustness of the reviews findings. This runs counter to previous findings on sourcing health effectiveness studies where additional searches designed to increase a review’s comprehensiveness have been limited in use because of the low quality of the research found [25].

We have shown that a small number of studies located from searching databases in addition to those specified in the original review protocol had a significant impact on the outcome of the final review. Although this case study is based upon 28 studies included in one review, the studies were from different subject disciplines, dispersed across a range of databases, and over a fifth were located from sources other than databases or search engines. In this case, a small number of studies were found in several places across large international bibliographic databases, and for other studies, the research records were scattered across a range of search sources over the disciplines of medicine, social science and education. At the outset of the systematic review it was anticipated that the literature would be widely dispersed. The original search protocol aimed to draw on a large sample of studies from a range of sources. It contained sources judged to potentially contain good sources of UK studies, notably: Applied Social Sciences Index and Abstracts (ASSIA), Bibliomap (EPPI-Centre's register of health promotion research), and HealthPromis (a UK Health Development Agency database that is no longer updated). ASSIA is an international social science database comprising of largely of journals published in the UK (46%) and in the USA (43%)[26]. The later searches of seven additional sources provided a greater UK focus to the search strategy. These contained UK studies not found by other means, rather than containing exclusively UK studies. For example, the International Bibliography of Social Sciences (IBSS) covers a large range of countries, and a majority (18%) of its source titles are published in the UK [27]. We cannot provide an accurate assessment of the time taken to undertake the additional searches. It involved a visit to another library to access one database; for some databases multiple simplified searches were undertaken owing to limited search functionality; and some databases were scanned at source, owing to restrictions in downloading the research records.

Although this case study is based on the results of searches used in the review, rather than what is actually present within the search source, we consider this justified as it is representative of the process of searching for a systematic review. The search strategies used for each database source were conceptually the same with variations depending on the functionality and volume of records. The database searches were intended to be sensitive, using a range of free text and controlled vocabulary terms, and this is demonstrated in Table 3 by the low precision values. Publication type has only been given a cursory consideration here, and when searching for specific types of research, the nature of the publication type may also have an impact in deciding where to search.

Searching for ‘views’ studies can involve considerable investment of resources. The low precision found from database sources is consistent with published findings, such as Shaw et al. [2] who report a precision of under four percent in database searching for qualitative studies. For such a small number of studies, it would not be sensible to use this case study for selecting one search source over another, but it is useful in increasing awareness of the types and range of sources that might be useful in limiting database selection bias. Search engines such as Google and Scirus are difficult to search comprehensively, but they can be used for supplementary searching to locate potentially useful studies. Response from contacting authors can be a good source, although this method has unpredictable outcomes. Forward citation chasing was not effective in yielding any new studies in this case; however, it was limited to Web of Science Cited Reference Search and in retrospect, additionally utilising a citation search database that covers non-journal reports and dissertations may have improved this. Our finding that a high proportion of studies (21% (n = 6)) were obtained only from non-database sources (as shown in Table 1) is similar to other studies of searching for qualitative research [12,13].

Identifying search sources for systematic reviews can be challenging, but could be improved with more knowledge-sharing between information specialists and researchers. There will always be limitations on how wide one is able to search, the number of strategies that can be employed to improve comprehensiveness and reduce database selection bias, and relevant studies could be missed. Making more informed choices of where to search can mitigate the effects of this. The EPPI-Centre is building on this case study and comparing search sources across a number of systematic views of people’s views [5].

Locating studies from a particular region is not just relevant to ‘views’ studies. A greater awareness of the value of searching a wider range of sources and the geographical slant of sources has contributed to the search protocol for other public health reviews within the EPPI-Centre. In undertaking other reviews we have identified other small databases and websites that could be of potential use. It can be challenging for the user to discover what is covered by a database or how publication sources are selected for inclusion, particularly where journals are partially indexed. In this case study, two included studies were from the Health Education Journal, and both were not picked up by database searches. Further investigation revealed partial indexing of this journal in electronic database sources and has provided a case for hand-searching of this journal in later reviews.

The impact of new technologies on the choice of database is another consideration. Some databases have had substantial investment in their IT infrastructure. PubMed, in particular has spawned a small ecosystem of supporting services which all build on and publish the PubMed dataset in different forms. GoPubMed offers semantic and other analyses of PubMed documents in an interface that few social science databases can match. HubMed offers an alternative interface and a highly useful ‘citation finder’ which enables users to copy and paste bibliographies into a text box that processes this into a list of citations with links to their PubMed records. PubMed itself offers an open application programming interface (API) that enables programmers to integrate searching PubMed into their applications. These new services are innovative and demonstrate the potential of modern IT technologies. However, they are becoming a source of potential bias, as the very availability and accessibility of these services promotes the PubMed dataset above smaller, regional databases.

## Conclusions

Our findings highlight the value in careful consideration of search sources in developing a search strategy, although searching more extensively does not guarantee locating more quality studies. Selecting sources on the basis of topic-coverage and study design is well-established, and we urge consideration of the geographical nature of database sources where appropriate. Policymakers, researchers and practitioners should be aware of the potential impact of where research literature is drawn from on the findings and relevancy of systematic reviews. This retrospective analysis demonstrates that ‘database selection bias’ can influence the outcome of a systematic review. It illustrates how the choice of search sources can increase geographical relevance. Despite aiming for comprehensiveness in literature searching, there are limitations in what can be found. A search protocol covering a range search sources, appropriate subject disciplines and geographical regions reduces the potential for missing important studies. This study has also demonstrated the value to be gained from obtaining external input into the review protocol from an advisory group. Owing to the lack of published research and case studies on both database selection bias and searching for views studies, there is a need for further dissemination and knowledge-sharing by information professionals and systematic reviewers on search sources for reviews.

## Competing interests

The authors declare that they have no competing interests.

## Authors’ contributions

CS, JK, AG and RR generated the concept and design of the study. CS analysed the search sources and RR analysed the impact of the studies on the systematic review. All authors participated in the development of the manuscript from its early stages. All authors contributed to and approved the final manuscript.

## Pre-publication history

The pre-publication history for this paper can be accessed here:

http://www.biomedcentral.com/1471-2288/12/55/prepub
